# Using a Household Food Inventory to Assess the Availability of Traditional Vegetables among Resettled African Refugees

**DOI:** 10.3390/ijerph13010137

**Published:** 2016-01-18

**Authors:** Catherine Gichunge, Shawn Somerset, Neil Harris

**Affiliations:** 1 School of Health Sciences, Mount Kenya University, P.O. Box 342, Thika 01000, Kenya; 2 School of Allied Health, Australian Catholic University, P.O. Box 456, Virginia, Brisbane 4001, Australia; shawn.somerset@acu.edu.au; 3 Population and Social Health Research Program, Menzies Health Institute Queensland, Griffith University, Gold Coast 4222, Australia; n.harris@griffith,edu.au

**Keywords:** household food inventory, African traditional vegetables, vegetable availability, vegetable intake, resettled African refugees, migration

## Abstract

A cross-sectional sequential explanatory mixed methods study was conducted among household food preparers to examine the association between home availability and consumption of traditional vegetables among resettled African refugees living in Queensland, Australia. Home availability of traditional African vegetables was associated with age, having a vegetable garden, employment status, and having a supermarket in the local neighborhood. Food preparers from homes with low vegetable availability were less likely to consume the recommended number of vegetable servings. Barriers faced in the food environment included language, lack of availability of traditional vegetables and lack of transport. All of these aspects contributed to the study findings that both individual and food environment characteristics may play a role in access to and availability of food and vegetable consumption of resettled refugees. Consumption of traditional foods among the resettled refugees continues post resettlement.

## 1. Introduction

Migration, whether local or international, forced or voluntary, invariably leads to a change of environment that migrants have to adapt to and how this is done may impact their health positively or negatively. Refugees who are forcibly removed from their countries inevitably find themselves seeking refuge in camps, villages or towns in countries socially, culturally and environmentally different from their own. The world refugee population has grown substantially over the past few decades [1] and protracted situations in countries like the Syrian Arab Republic, the Democratic Republic of Congo, Somalia and Sudan make it difficult for refugees to return to their homeland. This leads to resettlement in either a first or third country of asylum to enable them to lead a “normal” life while offering them protection. In 2014, the UNHCR resettled 73,008 refugees of which 26% were from Africa with those from Somalia, Sudan, the Democratic Republic of Congo and Eritrea [1].

African refugees invariably spend an extended time in camps where the main diet is derived from food aid [2] which may cushion them from consuming processed foods typical of the nutrition transition. However, it is well documented that upon resettlement refugees increase their consumption of highly processed foods and meats [3,4] and decrease their intake of fruits and vegetables [4]. As a result of these changes in dietary habits, many resettled African refugees develop a high BMI despite having a low BMI on arrival [5]. Similarly, diabetes a risk factor for cardiovascular diseases (CVD) has also been found to be high among resettled African refugees [6].

Difficulties in accessing food outlets that stock traditional foods in their new food environment may hinder the consumption of traditional foods [7,8,9]. Ease of access to food outlets stocking healthy food options is important as it enables healthy food choices. Less healthy household food supplies have dietary consequences for household members [10], as this may promote or hinder healthy eating. Associations have been reported between home availability and intake of fruits and vegetables [10,11], and fatty and unhealthy foods [10,12]. Within the home environment, underlying demographic factors such as household income, age, household composition, and education have been associated with food availability as well as vegetable and fruit intake [13,14].

Little is known about the home food environments of resettled African refugees. Insight into the foods available in their homes will provide a greater understanding of their home food environment and food habits, and further contribute to the development of appropriate intervention programs targeting food choices. Traditional African vegetables are unique to the African diet and hence it is important to determine their availability in the home and consumption upon resettlement. The aim of this study was to examine the association between household availability and consumption of traditional African vegetables among resettled African refugees residing in Southeast Queensland, Australia.

## 2. Experimental Section

This study used a cross sectional sequential explanatory mixed methods design to investigate the home availability of African vegetables among 71 resettled Burundian, Congolese and Rwandan refugees. Quantitative data were collected and analyzed prior to a qualitative interview guide being developed to explore and extend the quantitative findings.

### 2.1. Sample Recruitment

Purposive sampling was used to recruit participants for this study. As refugees are considered a difficult to reach population [15], participant recruitment occurred via several mechanisms. Participants were recruited from African churches, community meetings and settlement agencies. The various sources of recruitment allowed the researcher to maximise recruitment and diversity within the sample, as well as reduce sample selection bias. Nonetheless, the recruitment process was slow and data were collected between April 2012 and April 2013. Primary food preparers from households with children under 18 years were recruited. A total of 71 primary food preparers were recruited from 71 households. For their time and effort, each participant received a local supermarket grocery voucher valued at AUD $25. All participants provided written informed consent.

### 2.2. Data Collection

The research instruments were forward translated to Swahili from English. The translation was done by a professional translator and reviewed by the first author, with both being native speakers of Swahili and proficient in English. Both the translated and original versions of the instrument were pre-tested to ensure that the translation was interpreted accurately. Participants were interviewed in the language of their choice, English or Swahili. This was done to ensure that participants were comfortable and able to express themselves and most importantly their comments and opinions were not “lost” in translation.

#### 2.2.1. Quantitative Data

A researcher-administered questionnaire was used, comprising questions on demographics and socioeconomic characteristics, food environment and household food inventory (HFI). A predefined HFI shown in Table 1, consisting of 26 traditional African traditional vegetables, was used to assess the availability of these vegetables in the home. Community members from the African countries of interest were approached and asked to provide names of the traditional vegetables that they ate in their home countries. Vegetable names were provided in members’ native language, Swahili and, when known, the English name was also included. The Swahili names were then translated into English and the vegetable list and pictures were shown to the community members to ensure that there was a match between the vegetable names and pictures. This was done until the members confirmed that the pictures matched the foods on the list and the list was comprehensive. During data collection participants were asked if the foods on the lists were available in their homes and if one was not sure, they were asked to check their storage cabinets and fridge during the interview. For every positive response, a point was given, and the total sum of vegetable availability was used in the analysis with higher scores depicting greater availability. The vegetable availability score was categorized into two groups: low and high. Availability scores that were less than the mean were categorized as low, while those which were equal or greater than the mean were categorized as high. This scoring strategy is similar to that deployed in a study by Koui and Jago [16].

**Table 1:** Household food inventory.

Vegetable	Is This Vegetable Availabile in Your Home?	
	Yes	No
Amaranth		
Arrowroot leaves		
African eggplant leaves		
Bean leaves		
Beans (soft)		
Beans (dry)		
Black night shade		
Cassava leaves		
Cowpeas		
Cowpeas leaves		
Jute mellow		
Pumpkin leaves		
Sweet potato leaves		
Taro leaves		
Spider plant		
African egg plant		
Arrow root		
Cassava		
Green bananas (plantains)		
Pumpkin		
Sweet potato (white)		
Sweet potato (yellow)		
Taro (small)		
Taro (big)		
Indigenous potato/Country potato		
Yams		

For this study the neighborhood food environment was assessed by asking participants if there was a farmer’s market or supermarket in their neighborhood. Reponses to these questions were dichotomized as “yes or no”. The neighborhood was defined as the suburb in which the participant resided. Participants were also asked if they had a vegetable garden. The intake of traditional African vegetables was assessed using a food frequency questionnaire (FFQ) with items that were on the HFI. Participants were asked to indicate their intake frequencies by selecting from nine frequencies that ranged from “never” to “more than once a day”. The frequencies were converted to daily frequencies using the conversions shown in Table 2. The portion size for each item was determined using household measures provided by the researcher to promote uniformity. The daily servings for each item on the FFQ were calculated by multiplying the reported portion size and intake of each item as done in a previous study [17]. The total vegetable intake was then coded to be consistent with current Australian Nutrition Guidelines on serving sizes and recommended intake of vegetables for adults: 0–2 servings, 3–4 servings and 5 and above servings [18].

**Table 2:** Food frequency conversions.

Frequencies	Conversions
Never	0.0
Less than once per month	0.01
1–3 times per month	0.07
Once per week	0.14
2–3 times per week	0.36
4–5 times per week	0.64
6 times per week	0.86
Once per day	1
More than once per day	2

#### 2.2.2. Qualitative Data

After analysis of the quantitative data, qualitative data were collected using in-depth interviews to explore the findings of the quantitative results. Fifteen participants who had completed the quantitative survey were purposively selected for the qualitative interviews. Three questions were developed to explore the quantitative results: Where do you get the vegetables? Why do you have these vegetables in your home? What problems do you encounter when sourcing your traditional vegetables in your neighborhood? The interviews lasted approximately 45 min each and were audio recorded (with participant consent) for subsequent verification of written notes.

### 2.3. Data Analysis

The Statistical Package for the Social Sciences (SPSS) version 20 (SPSS Inc, Chicago, IL, USA) was used for statistical analysis. Chi square analysis was used to determine associations between demographic and socioeconomic characteristics and food environment with home vegetable availability. Logistic regression was conducted to assess associations of home availability of African vegetables with vegetable intake adjusting for demographic and food environment characteristics that were significant in the bivariate analysis. The statistical significance level was set at *p* < 0.05. Recordings of the qualitative interviews were transcribed verbatim and the transcripts were read several times to understand their content. The transcripts were reviewed as part of a data collection and analysis cycle to establish if there was a need to collect additional information to aid in interpreting the findings. This was done until saturation was achieved with no new or different information emerging from the interviews. Using text analysis, a line by line analysis was conducted on the transcripts to identify categories. These categories were reviewed to ensure that they provided responses to questions on the interview guide.

### 2.4. Ethical Approval

This study was approved by a University Human Research Ethics Committee (PBH/36/11/HREC). All participants provided written informed consent.

## 3. Results and Discussion

### 3.1. Results

#### 3.1.1. Quantitative Results

The primary food preparer from each of the 71 households was surveyed. The average household size was 5.4 ± 2.2 with an average of 1.8 ± 0.7 adults and 3.6 ± 1.94 children. The sample was predominately female (88.7%) and from Burundi (74.6%). The HFI revealed that participants had a mean of 5.4 ± 2.6 types of African vegetables in their homes at the time of audit. In the bivariate analysis shown in Table 3, the availability of African vegetables in the home was significantly associated with age, employment, having a supermarket in the neighborhood, and having a vegetable garden (*p* < 0.05). Those who were older, employed, engaged in vegetable gardening and had a supermarket in their neighborhood, reported high availability of traditional African vegetables in the home.

**Table 3:** Associations between socio-demographic and food environment characteristics with home availability of traditional vegetables.

Variable	HFI African Vegetable		
	Low *n* (%)	High *n* (%)	*p* Value *
Age			0.001
<30	17 (85.5)	3 (15)	
30 to 39	13 (44.8)	16 (55.2)	
≥40	5 (22.7)	17 (77.3)	
Education level			0.060
Primary education and below	13 (38.2)	22 (61.8)	
High school education and above	22 (59.5)	15 (40.5)	
Employment status			0.005
Unemployed	29 (60.4)	19 (39.6)	
Employed	5 (23.8)	16 (76.2)	
Annual household income			0.506
<$20,000	10 (50)	10 (50)	
$20,000–30,000	15 (55.6)	12 (44.4)	
>$30,001	9 (39.1)	14 (60.9)	
Duration in Australia			0.086
Less than 5 years	14 (63.6)	8(36.4)	
5 years and more	21 (42.9)	28 (57.1)	
Ability to speak English well			0.144
No	17 (42.5)	23 (57.5)	
Yes	18(58.1)	13 (41.9)	
Supermarket in neighborhood			0.032
No	5 (27.8)	13 (72.2)	
Yes	30 (56.6)	23 (43.4)	
Farmers’ market in neighborhood			0.883
No	18 (48.6)	19 (51.4)	
Yes	15 (46.9)	17 (53.1)	
Grow own vegetables			0.001
No	25 (67.6)	12 (32.4	
Yes	10 (29.4)	24 (70.6)	

* Chi square test

In the logistic regression analysis participants who reported low home availability of traditional vegetables were 0.19 (95% CI 0.057–0.634; *p* = 0.007) times more likely to consume 0–2 vegetable servings compared to those from households with high vegetable availability (Table 4).

**Table 4:** Associations between home availability and intake of traditional vegetables.

Varaible	OR	95% CI	*p* Value
Vegetable Intake			
5 and above servings (ref)	1.00		
0–2 servings	0.19	0.057–0.634	0.007
3–4 servings	0.813	0.226–3.209	0.813

#### 3.1.2. Qualitative Results

Fifteen participants from the quantitative study were purposively selected to participate in the qualitative study. Table 5 shows their socio demographic and neighborhood food environment characteristics.

**Table 5:** Characteristics of participants who took part in the qualitative interview.

ID	Age	Employed	Number of Children	Food Environment (Supermarket and Farmers’ Market in Neighborhood) *	Growing Own Vegetables (Garden)
1	40	Yes	5	SM, FM	Yes
2	34	No	5	SM, FM	Yes
3	32	No	4	SM, FM	Yes
4	40	Yes	3	None	No
5	36	Yes	4	None	Yes
6	37	No	3	SM	No
7	37	No	5	None	Yes
8	40	No	4	None	Yes
9	27	No	2	SM, FM	Yes
10	52	No	2	SM, FM	Yes
11	43	Yes	6	None	Yes
12	31	No	2	SM, FM	No
13	33	No	5	SM, FM	Yes
14	31	No	1	SM, FM	No
15	35	No	2	SM, FM	No

* SM = supermarket, FM = farmers’ market.

##### Sources of Vegetables

Participants reported that they sourced their traditional vegetables from a variety of outlets that included supermarkets, farmers’ markets and ethnic grocery shops. Nine of the 15 respondents had supermarkets and a farmers’ market in their neighborhood while one participant only had a supermarket in her neighborhood. However, five participants did not have a supermarket or farmers’ market in their neighborhood. Compared to supermarkets and ethnic grocery stores, farmers’ markets were the preferred food outlet in terms of availability and cost by all of the 15 participants.

If you do not go to the Sunday market (farmers’ market) you will not get African vegetables. ID 12

Some of the vegetables are available in the African and Indian shops but they are cheaper and abundant at the Sunday market (farmers’ market). ID 15

Community or home vegetable gardens were identified as an alternative source of African vegetables. Ten of the 15 participants who engaged in vegetable gardening reported that African vegetables were available and accessible at no expense.

I used to buy vegetables (amaranth) but now I do not buy. I harvest from my garden. ID 5

##### Reasons for Stocking Traditional Vegetables in the Home

Four factors that influenced the availability of vegetables in the participants’ homes were identified: vegetables were perceived to be healthy foods; family preferences; food preparation skills; and the traditional foods were familiar and more filling. All 15 participants reported that vegetables were healthy, hence the reason they were available in their homes. According to these participants, vegetables were fat free and provided the body with important nutrients.

Vegetables are healthy. They have no oil that can give you problems. ID 6

They (vegetables) are healthy. Even when we were in Africa we were told they are good. Like cassava leaves adds blood to the body. Vegetables are good for health. ID 2

Home vegetable availability was influenced by the participant’s preferences. All the participants reported that they preferred their traditional vegetables, and that is why they were available in their homes.

I can not go for 3 days without eating beans. I like to eat beans. Beans, all vegetables. I cook beans and green bananas. I like them a lot. I also eat cassava. ID 10

Five of the participants reported that their spouses’ preferences for the traditional vegetables contributed to the availability of these vegetables in the home.

He (husband) is like me. He does not like fatty foods. We like to cook like Africa. We cook beans and add pumpkins and make stew. We also make beans and cassava stew. ID 7

Children’s preference for traditional vegetables was reported by 11 participants. Cassava leaves were mentioned by 11 participants as the traditional vegetable that their children liked the most. One participant reported that her children liked cassava leaves and amaranth.

My children like sombe (cassava leaves) and lengalenga (amaranth). ID 3

One participant reported that her children had no problem eating the African vegetables when they were in Africa, but that changed when they arrived in Australia. The children now ate small quantities of the African vegetables and developed a preference for other foods found in Australia. She however continues to give them the traditional vegetables.

My children were eating our food in Africa but they do not want it. If you cook chips and chicken they like it. They eat small quantities of sombe and lengalenga. ID 13

Two of the 15 participants reported forcing their children to eat vegetables. These two participants were aware of the nutritional benefits of vegetables and wanted their children to eat vegetables. One of these two participants reported that her child liked meat and she had to force her to eat vegetables. For these two participants their vegetable availability was influenced by their desire to ensure that their children had access to healthy foods.

… She does not like it at all. She does not like the vegetables I cook. She just likes meat, meat, meat. I force her to eat vegetables. I have to sit next to her. She will chew one spoonful for five minutes. ID 14

Traditional vegetables were purchased by all the participants as they were the foods that the participants were accustomed to. Participants reported purchasing these vegetables as they were familiar foods, foods that they were brought up eating, and the foods that their parents ate. They considered these foods a part of their lives.

I can say culture. I buy stuff I used to eat in my country. We are still eating the same food we have not changed our meal style. That is why when I go shopping I focus on what we used to eat, what the kids like or the whole family likes. I am happy as I find all the stuff that I used to buy in my country. ID 1

Other than being familiar, these are the vegetables they know how to prepare. One participant reported that she once bought Brussels sprouts but she threw them away as she did not know how to cook them. Although all the participants had incorporated new foods in their diet, there was a preference for traditional vegetables. One participant reported that these traditional foods were more filling compared to Australian foods.

Respondent: When I eat them (traditional vegetables) I get very satisfied but when I eat the foods from here (shrugs shoulders).

Interviewer: “What foods do not make you satisfied?”

Respondent: Spaghetti (pause). Even rice. When I eat rice I do not get satisfied. But when I cook my beans and its leaves I can eat ‘til I burst. I really like it. ID 11

##### Problems Encountered in the Food Environment

Problems faced by the participants when sourcing their traditional vegetables were grouped into two categories: language barriers and traveling to other neighborhoods. Four participants reported that they could not read English which made shopping especially in the supermarkets a challenge. Unlike in their home country where they had shopped in open markets, the Australian shops arrange foods in aisles with signs indicating the foods that are available in each aisle. For those who could not read English, this was a problem.

Shops here are different. At home we used to shop in the market. Here things are on the shelves and aisles and it takes long to know where the food is. And you know you cannot ask where things are all the time as you are expected to read. ID 3

Lack of preferred vegetables in the neighborhood food outlets was also a challenge which forced six participants to visit several shops and travel to other neighborhoods. Although one participant reported that a non-petrol convenience shop was available in his neighborhood, it did not sell any traditional foods.

The shop in my neighborhood does not sell African food. They sell wazungu (white people) food, food for people who have been born here. We prefer our food. Like beans, there are no beans there, things like ugali flour, cassava, cassava leaves. So I have to go to (name of neighborhood) and (name of neighborhood). ID 5

Lack of transportation was a challenge identified by six participants that limited their ability to access their preferred food outlets in other neighborhoods. Lack of reliable transport meant that these participants had to use public transport which limited the amount of foods they purchased as well as the food outlets visited. This may also have influenced the availability or at least variety of vegetables in the home.

I am not able to go to the Sunday market (farmers’ market) as it is in (name of neighborhood) and I do not have a car. It is very difficult to travel on the train with food and children. Even when I go with the train, the market is far from the train station. ID 4

### 3.2. Discussion

This study found associations between the home availability of traditional African vegetables with participants’ age, employment status, having a vegetable garden and the availability of a supermarket in the neighborhood. The association between age and home availability of African vegetables may be linked with older participants’ preferences for their traditional foods while in post resettlement [8]. One participant expressed this by saying: “*We have done this for a long time. We do not want to change our culture. We want to keep our culture, eating the same stuff we were eating before*”. Culture is a determinant of food habits [19] and food is central to individual identity [20]. Referring to their traditional foods as “our food” or “my food” is an indication that they perceive their traditional foods as part of their cultural and ethnic identity, findings that have been reported among other resettled refugees [8,9].

Those living in households with low availability of traditional vegetables were less likely to consume the recommended number of vegetable servings. This low vegetable intake compares to that reported among the general Australian population, which shows that the majority of the population do not consume the recommended number of vegetable servings [21]. A large percentage of those who were unemployed reported low vegetable availability which may be associated with the relatively high cost of traditional African vegetables [8,9]. As home vegetable availability is associated with vegetable intake [10,22], a potential intervention strategy to enhance vegetable intake resettled refuge is to encourage them to stock their homes with vegetables, although the impact of this remains to be tested.

There was an association between the home availability of traditional vegetables and having a vegetable garden. Those who gardened reported high availability of traditional vegetables; this may be because they had planted their traditional vegetables. Food gardening provides access to fresh foods and has been associated with an increase in the consumption of fruits and vegetables [23,24]. Resettled refugees have reported that traditional foods are expensive and difficult to locate [8,9] hence having a food garden offers a cost-effective means to increase availability of traditional foods in the home.

Despite there being no association between home availability of traditional vegetables and the availability of farmers’ markets in the local neighborhood, participants reported shopping in farmers’ markets as they were perceived to be less expensive with a wide variety of fresh traditional foods. Nevertheless there was an association between the home availability of vegetables and having a supermarket in the local neighborhood. Neighborhood availability of supermarkets has been associated with increased intake of fruits and vegetables [25]. Participants also reported purchasing their traditional vegetables from ethnic stores. This may also have contributed to the availability of these foods in the home even though the association was not measured. Hence, refugees having access to a variety of outlets that stock their traditional foods could provide them with more opportunities to make healthy foods choices.

Participants reported purchasing vegetables that their children preferred, which may have increased the availability of these vegetables in the home. Children’s consumption of vegetables and fruits has been associated with the availability of these foods in the home [11]. However, one participant reported her children no longer preferred the traditional foods they ate in Africa but instead favored the highly processed foods available in their new environment. This finding is consistent with other studies on refugee children and their food choices [26,27]. Parents are important advocates for promoting and improving consumption of vegetables and other healthy foods among their children [11] thus, encouraging resettled refugees to continue stocking their homes with these healthy traditional vegetables as this may provide their children with healthy food options leading to nutritional health.

Language was noted as a problem when shopping for foods including vegetables in enclosed shops, supermarkets and grocery stores. This has previously been observed among resettled refugees and immigrants [28,29]. These language barriers may discourage participants from visiting certain shops to purchase vegetables, which may lead to a reduced vegetable intake or lack of vegetable intake. Other previously identified challenges that were confirmed in the present study included lack of traditional foods in their neighborhoods of residence [7,9], and having to visit several shops to source preferred traditional foods. A lack of transport hinders access to healthy, affordable and culturally relevant vegetables, especially if individuals have to travel outside their neighborhood to purchase them, which in turn, influences the availability of foods in the home.

Importantly, results from this study confirm previous observations that consumption of traditional foods among resettled refugees continues well beyond resettlement [3,9,17,30,31]. This pattern is in part because of perceptions that traditional foods are healthy and more filling [9,30,31,32] than the new foods that resettled refugees encounter in Australia and other highly industrialized countries. Research shows that these traditional vegetables have nutritional benefits and are superior to the processed foods and meats encountered upon resettlement, a sentiment that has significant health promotion potential [33].

This study has several limitations. The sample size was small (*n* = 71) and almost homogenous as most of the participants were from Burundi, with fewer participants from Rwanda and the Congo. This limits the generalizability of the study findings. It may be possible that nationality/ethnicity impacts home food availability, although this was not measured in this study. The HFI used in this study reported on the availability of traditional vegetables in the homes but did not report on the actual quantities available. Vegetable availability was only assessed at one point in time with data collected over autumn, winter and spring of 2012. The study was not able to take into account seasonal variations as well as variations in household food supplies and socio-demographic changes such as household composition and income. Conducting several household vegetable inventories could show changes in household food supplies over time. Self-reporting on the FFQ may have overestimated vegetable consumption since vegetables are promoted as a healthy food [34]. In addition, the present study found cross-sectional associations between various potential determinants and traditional African vegetable consumption. The temporality of these associations remains to be tested through longitudinal study designs. Despite these limitations, the study has several strengths. Data were collected in the home; hence participants were able to check their food storage when reporting on the availability of the foods listed on the HFI. This HFI was easy to administer and included culturally preferred foods. This study contributes to the limited literature around household food availability among resettled refugees and immigrants [12,35].

## 4. Conclusions

This study provides an insight into the home availability of traditional vegetables among resettled African refugees and associated individual and food environment characteristics. Such information is useful in the development of health and nutrition promotion programs for similar population groups that have moved to a new and unfamiliar food environment. An emerging trend within the study was the lower vegetable intake among participants of households with low vegetable availability, supporting findings that home vegetable availability influences vegetable intake. Food preparers, such as the participants in this study, play a pivotal role as their food habits influence and predict foodhabits of their family members, especially children.

Interestingly, this study suggests that resettled African refugee food preparers retain preferences for their traditional vegetables and foods. However, this population has difficulties navigating their new food environment associated with language, transport and location of food outlets that may be hindering access to and consumption of these preferred traditional vegetables and foods. The research suggests that interventions that work with these resettled refugee communities need to facilitate better access to traditional vegetables such as community food gardening, as this would be of great cultural and nutritional value to these communities. Future studies should be longitudinal so as to provide more detailed information on changes of home vegetable availability and vegetable intake of both food preparers and children over time, taking into consideration seasonal changes as well as changes on the household socio-demographic characteristics.

## References

[1] UNHCR UNHCR Refugee Resettlement Trends 2015. http://www.unhcr.org/559e43ac9.html.

[2] World Food Programme (WFP) World Food Programme Food Basket. http://www.wfp.org/nutrition/WFP-foodbasket.

[3] Burns C. (2004). Effect of migration on food habits of Somali women living as refugees in Australia. Ecol. Food Nutr..

[4] Dharod J.M., Croom J., Sady C.G., Morrell D. (2011). Dietary intake, food security, and acculturation among Somali refugees in the United States: Results of a pilot study. J. Immigr. Refug. Stud..

[5] Guerin P., Elmi F., Corrigan C. (2007). Body composition and cardiorespiratory fitness among refugee Somali women living in New Zealand. J. Immigr. Minor. Health.

[6] Renzaho A.M.N., Bilal P., Marks G.C. (2013). Obesity, type 2 diabetes and high blood pressure amongst recently arrived Sudanese refugees in Queensland, Australia. J. Immigr. Minor. Health.

[7] Jacobus M.V., Jalali R. (2011). Challenges to food access among Lewiston’s African immigrants. Maine Policy Rev..

[8] Patil C.L., McGown M., Nahayo P.D., Hadley C. (2010). Forced migration: Complexities in food and health for refugees resettled in the United States. NAPA Bull..

[9] Gichunge C., Kidwaro F. (2014). Utamu Wa Afrika (the sweet taste of Africa): The vegetable garden as part of resettled African refugees’ food environment. Nutr. Diet..

[10] Fulkerson J.A., Nelson M.C., Lytle L., Moe S., Heitzler C., Pasch K.E. (2008). The validation of a home food inventory. Int. J. Behav. Nutr. Phys. Act..

[11] Wyse R., Wolfenden L., Bisquera A. (2015). Characteristics of the home food environment that mediate immediate and sustained increases in child fruit and vegetable consumption: Mediation analysis from the healthy habits cluster randomised controlled trial. Int. J. Behav. Nutr. Phys. Act..

[12] Satia J.A., Patterson R.E., Kristal A.R., Hislop T.G., Pineda M. (2001). A household food inventory for North American Chinese. Public Health Nutr..

[13] Pollard J., Greenwood D., Kirk S., Cade J. (2001). Lifestyle factors affecting fruit and vegetable consumption in the UK women’s cohort study. Appetite.

[14] Giskes K., Turrell G., Patterson C., Newman B. (2002). Socio-economic differences in fruit and vegetable consumption among Australian adolescents and adults. Public Health Nutr..

[15] Sulaiman-Hill C., Thompson S. (2011). Sampling challenges in a study examining refugee resettlement. BMC Int. Health Hum. Rights.

[16] Koui E., Jago R. (2008). Associations between self-reported fruit and vegetable consumption and home availability of fruit and vegetables among Greek primary-school children. Public Health Nutr..

[17] Gichunge C., Harris N., Tubei S., Somerset S., Lee P. (2015). Relationship between food insecurity, social support, and vegetable intake among resettled African refugees in Queensland, Australia. J. Hunger Environ. Nutr..

[18] National Health and Medical Research Council (2013). Australian Dietary Guidelines.

[19] Opare-Obisaw C., Fianu D.A.G., Awadzi K. (2000). Changes in family food habits: The role of migration. J. Consum. Stud. Home Econ..

[20] Fischler C. (1988). Food, self and identity. Soc. Sci. Inf..

[21] Australia Bureau of Statistics Profiles of Health, Australia, 2011–2013: Daily Intake of Fruit and Vegetable. http://www.abs.gov.au/ausstats/abs.

[22] Cullen K.W., Baranowski T., Owens E., Marsh T., Rittenberry L., de Moor C. (2003). Availability, accessibility, and preferences for fruit, 100% fruit juice, and vegetables influence children’s dietary behavior. Health Educ. Behav..

[23] Alaimo K., Packnett E., Miles R.A., Kruger D.J. (2008). Fruit and vegetable intake among urban community gardeners. J. Nutr. Educ. Behav..

[24] Litt J.S., Soobader M.J., Turbin M.S., Hale J.W., Buchenau M., Marshall J.A. (2011). The influence of social involvement, neighborhood aesthetics, and community garden participation on fruit and vegetable consumption. Am. J. Public Health.

[25] Moore L.V., Diez Roux A.V., Nettleton J.A., Jacobs D.R. (2008). Associations of the local food environment with diet quality—A comparison of assessments based on surveys and geographic information systems. Am. J. Epidemiol..

[26] Trapp M. (2010). What’s on the Table: Nutrition programming for refugees in the United States. NAPA Bull..

[27] Patil C., Hadley C., Nahayo P. (2009). Unpacking dietary acculturation among new Americans: Results from formative research with African refugees. J. Immigr. Minor. Health.

[28] Vahabi M., Damba C. (2013). Perceived barriers in accessing food among recent Latin American immigrants in Toronto. Int. J. Equity Health.

[29] Hadley C., Patil C.L., Nahayo D. (2010). Difficulty in the food environment and the experience of food insecurity among refugees resettled in the United States. Ecol. Food Nutr..

[30] Vue W., Wolff C., Goto K. (2011). Hmong food helps us remember who we are: Perspectives of food culture and health among Hmong women with young children. J. Nutr. Educ. Behav..

[31] Pereira C.A.N., Larder N., Somerset S. (2010). Food acquisition habits in a group of African refugees recetly settled in Australia. Health Place.

[32] Franzen L., Smith C. (2009). Acculturation and environmental change impacts dietary habits among adult Hmong. Appetite.

[33] Oniang’o R.K., Mutuku J.M., Malaba S.J. (2003). Contemporary african food habits and their nutritional and health implications. Asia Pac. J. Clin. Nutr..

[34] Miller T., Abdel-Maksoud M., Crane L., Marcus A., Byers T. (2008). Effects of social approval bias on self-reported friut and vegetable consumption: A randomized controlled trial. Nutr. J..

[35] Hearst M.O., Fulkerson J.A., Parke M., Martin L. (2012). Validation of a home food inventory among low-income Spanish- and Somali-speaking families. Public Health Nutr..

